# Threshold Digraphs

**DOI:** 10.6028/jres.119.007

**Published:** 2014-05-20

**Authors:** Brian Cloteaux, M. Drew LaMar, Elizabeth Moseman, James Shook

**Affiliations:** 1 National Institute of Standards and Technology, Gaithersburg, MD 20899; 2 The College of William and Mary, Williamsburg, VA 23185

**Keywords:** digraph realizations, Fulkerson-Chen theorem, threshold digraphs

## Abstract

A digraph whose degree sequence has a unique vertex labeled realization is called threshold. In this paper we present several characterizations of threshold digraphs and their degree sequences, and show these characterizations to be equivalent. Using this result, we obtain a new, short proof of the Fulkerson-Chen theorem on degree sequences of general digraphs.

## 1. Introduction

Creating models of real-world networks, such as social and biological interactions, is a central task for understanding and measuring the behavior of these networks. A usual first step in this type of model creation is to construct a digraph with a given degree sequence. We examine the extreme case of digraph construction where for a given degree sequence there is exactly one digraph that can be created.

What follows is a brief introduction to the notation used in the paper. For notation not otherwise defined, see Diestel [1]. We let *G* = (*V, E*) be a digraph where *E* is a set of ordered pairs called arcs. If (*v*, *w*) ∈ *E,* then we say *w* is an out-neighbor of *v* and *v* is an in-neighbor of *w*. We notate the out-degree of a vertex *v* ∈*V* by $d_{G}^{+}(v)$ and the in-degree as $d_{G}^{{}^{-}}(v)$, suppressing the subscript when the underlying digraph is apparent from context.

Given a sequence $\alpha =((\alpha_{1}^{+},\alpha_{1}^{{}^{-}}),\ldots ,(\alpha_{n}^{+},\alpha_{n}^{{}^{-}}))$ of integer pairs we say that *α* is **digraphical** if there is a digraph *G* = (*V*, *E*) with *V* = {*v*_1_,…,*v_n_*} and $d^{+}(v_{i})=\alpha_{i}^{+},d^{-}(v_{i})=\alpha_{i}^{{}^{-}}$. We call such *G* a **realization** of *α*. An integer pair sequence *α* is in **positive lexicographical order** if $\alpha_{i}^{+}\geq \alpha_{i+1}^{+}$ with $\alpha_{i}^{{}^{-}}\geq \alpha_{i+1}^{{}^{-}}$ when $\alpha_{i}^{+}=\alpha_{i+1}^{+}$.

We are interested in the degree sequences that have unique vertex labeled realizations and the digraphs that realize them. Theorem 1 in Sec. 2 presents several characterizations of this type of degree sequence and its realization. We then show these characterizations to be equivalent. One of the characterizations is previously unpublished, and allows for a much shorter proof of the equivalence of the two known characterizations as well as proving the final characterization which appears without proof in the literature. In Sec. 3, we use Theorem 1 to obtain a new short proof of the Fulkerson-Chen theorem on degree sequences of general digraphs. We end by presenting some applications in Sec. 4.

## 2. Threshold Digraph Characterization

**Fig. 1:**
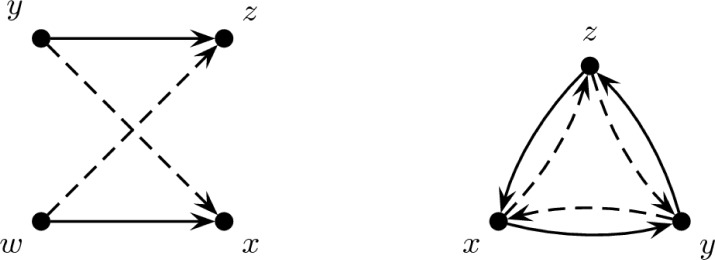
A 2-switch (left) and an induced directed 3-cycle (right). Solid arcs must appear in the digraph and dashed arcs must not appear in the digraph. If an arc is not listed, then it may or may not be present.

In the existing literature [2], the characterization of the unique realization of a degree sequence is in terms of forbidden configurations. The two forbidden configurations are the 2-switch and the induced directed 3-cycle. A **2-switch** is a set of four vertices *w*, *x*, *y*, *z* so that (*w*, *x*) and (*y*, *z*) are arcs of *G* and (*w*, *z*), (*y*, *x*) are not. An **induced directed 3-cycle** is a set of three vertices *x*, *y*, *z* so that (*x*, *y*),(*y*, *z*),(*z*, *x*) are arcs but there are no other arcs among the vertices. Replacement of the arcs in these configurations with the arcs that are not present yields another digraph with the same degrees, both in and out, so any degree sequence of a digraph with these configurations has multiple realizations. These configurations are pictured in Fig. 1.

Our main theorem shortens the existing proofs by showing the equivalence of our characterization (Condition 3 in Theorem 1) to known characterizations.

**Theorem 1.** *Let G be a digraph and A* = [*a_ij_*] *an adjacency matrix of G. Define* $\alpha_{i}^{+}=\sum_{j=1}^{n}a_{ij}$ *and* $\alpha_{j}^{{}^{-}}=\sum_{i=1}^{n}a_{ij}$*. Suppose that the vertices v*_1_,…,*v_n_ of G are ordered so that* $d^{+}(v_{i})=\alpha_{i}^{+},d^{-}(v_{i})=\alpha_{i}^{{}^{-}}$ *and the degree sequence* $\alpha =((\alpha_{1}^{+},\alpha_{1}^{{}^{-}}),\ldots ,(\alpha_{n}^{+},\alpha_{n}^{{}^{-}}))$ *of G is in positive lexicographic order. The following conditions are equivalent:*

1. G is the unique labeled realization of the degree sequence α.
2. There are no 2-switches or induced directed 3-cycles in G.
3. *For every triple of distinct indices i, j and k with i* < *j, if a_ik_* = 1,* then a_jk_* = 1.
4. *The Fulkerson-Chen inequalities are satisfied with equality. In other words, for* 1 ≤ *k* ≤ *n,*
  (1) $$\sum_{i=1}^{k}\min(\alpha_{i}^{{}^{-}},k-1)+\sum_{i=k+1}^{n}\min(\alpha_{i}^{{}^{-}},k)=\sum_{i=1}^{k}\alpha_{i}^{+}.$$

*Proof.* The equivalence of conditions 1 and 2 has been shown previously [2]. For this proof, we only need to show the implication 1 ⇒ 2, which is shown by the contrapositive: if there were a 2-switch or an induced directed 3-cycle in *G*, then we can form another graph *G*′ on the same degree sequence so *G* does not have a unique realization. Notice that this implication does not require positive lexicographic order.

2 ⇒ 3 (Proof by contrapositive: ¬3 ⇒ ¬2.) Let *n* ≥ 3 and *i*, *j*, *k* distinct indices so that *i* < *j*, *a_jk_* = 1 and *a_ik_* = 0. Let *l* ∉ {*i, j*, *k*}, if such an index exists, and note that what follows holds vacuously if *n* = 3 and no such *l* exists. For this *l*, if *a_il_* = 1 and *a_jl_* = 0, then the arcs (*v_i_*, *v_l_*) and (*v_j_*, *v_k_*) form a 2-switch. Otherwise, define *κ* (*x*, *y*) =|{*l* ∉{*i*, *j*,*k*}| *a_il_* = *x*,*a_jl_* = *y*}| for *x*, *y* ∈{0,1} and notice that *κ* (1,0) = 0. Thus, $\alpha_{i}^{+}=a_{ij}+\kappa (1,1)$ and $\alpha_{j}^{+}=a_{ji}+1+\kappa (1,1)+\kappa (0,1)$. Since $\alpha_{i}^{+}\geq \alpha_{j}^{+}$, we have *a_ij_* ≥ *a_ji_* +1+*κ* (0,1) so *a_ij_* = 1, *a_ji_* = 0, *κ* (0,1) = 0 and $\alpha_{i}^{+}=\alpha_{j}^{+}$.

Now we consider the in-degree of *v_i_* and *v_j_*. Since *a_ij_* = 1, *a_ji_* = 0 and $\alpha_{i}^{{}^{-}}\geq \alpha_{j}^{{}^{-}}$ there must be a vertex *v* so that (*v*, *v_i_*) is an arc and (*v*, *v_j_*) is not an arc. If *v* = *v_k_*, then the vertices *v_i_*, *v_j_* and *v_k_* form an induced directed 3-cycle. Otherwise, set and *v* = *v_l_* and consider *a_lk_*. If *a_lk_* = 0, then the arcs (*v_l_*, *v_i_*) and (*v_j_*, *v_k_*) form a 2-switch. Otherwise, *a_lk_* = 1 and the arcs (*v_l_*, *v_k_*) and (*v_i_*, *v_j_*) form a 2-switch.

3 ⇒ 4. Let *A_k_* be the *k* × *n* submatrix of *A* with only the first *k* rows. We count the number of ones in this matrix by rows to obtain $\sum_{i=1}^{k}\alpha_{i}^{+}$ and note that if *j* ≤ *k* there are $\sum_{i=1}^{k}a_{ij}=\min(\alpha_{j}^{{}^{-}},k-1)$ ones in column *j* and if *j* > *k* there are $\sum_{i=1}^{k}a_{ij}=\min(\alpha_{j}^{{}^{-}},k)$ ones in column *j*, then the count of ones by column is $\sum_{j=1}^{k}\min(\alpha_{j}^{{}^{-}},k-1)+\sum_{j=k+1}^{n}\min(\alpha_{j}^{{}^{-}},k)$. Thus $\sum_{j=1}^{k}\min(\alpha_{j}^{{}^{-}},k-1)+\sum_{j=k+1}^{n}\min(\alpha_{j}^{{}^{-}},k)=\sum_{i=1}^{k}\alpha_{i}^{+}$, as desired. Notice that this implication does not require positive lexicographic order.

4 ⇒ 1. Assume that *α* is in positive lexicographic order and that we have equality in the Fulkerson-Chen inequalities. We will form the adjacency matrix *A* one column at a time. Let $c(i,k)=|\{j\leq k|a_{ji}=1\}|$, the number of ones in the first *k* rows of the *i^th^* column. For any *k*, we have that the number of ones in the submatrix *A_k_* is given by $\sum_{i=1}^{k}\alpha_{i}^{+}=\sum_{i=1}^{n}c(i,k)$. Notice that for each *i* and *k* we have

(2) $$c(i,k)\leq \{\begin{array}{ll} \min(\alpha_{i}^{{}^{-}},k-1) & i\leq k\\ \min(\alpha_{i}^{{}^{-}},k) & i>k. \end{array}$$

Since we have equality in the Fulkerson-Chen conditions, we must also have equality for each *c*(*i*,*k*). In particular, considering column *i*, if $\alpha_{i}^{{}^{-}}\geq i$, then let $k=\alpha_{i}^{{}^{-}}+1$. Notice that $c(i,k)=\min(\alpha_{i}^{{}^{-}},k-1)=\alpha_{i}^{{}^{-}}$, and, since *a_ii_* = 0, there are only $\alpha_{i}^{{}^{-}}$ positions for the ones in this column of *A_k_*. Therefore, *a_ji_* = 1 for every *j* ≠ *i* and $j\leq k=\alpha_{i}^{{}^{-}}+1$. This is the number of ones in this column so the rest are zeros. If $\alpha_{i}^{{}^{-}}<i$, let $k=\alpha_{i}^{{}^{-}}$. Again, $c(i,k)=\min(\alpha_{i}^{{}^{-}},k)=\alpha_{i}^{{}^{-}}$ and there are only $\alpha_{i}^{{}^{-}}$ positions for ones in this column of *A_k_*. Thus, *a_ji_* = 1 for every *j* ≤ *k* and *a_ji_* = 0 for every *j* > *k*. Each of these choices was forced, so every arc in *G* is forced and *G* is the unique realization of *α*. The only place that this requires positive lexicographic order is the set-up: to satisfy the Fulkerson-Chen conditions with equality requires *α* to be in positive lexicographic order.

We call any digraph that satisfies these conditions **threshold**. This definition generalizes the well-studied concept of threshold graphs [3].

As mentioned above, Rao, Jana, and Bandyopadhyay [2] showed the equivalence of conditions 1 and 2 in the context of Markov chains for generating random zero-one matrices with zero trace. Condition 4 appears in the literature (for example, Berger [4] states this as the definition of threshold digraphs), but we cannot find a proof of its equivalence to the first two conditions. Condition 3 is similar to the criteria of Berger [5, 6] stated without proof in the context of corrected Ferrers diagrams.

There are two places where the order of *α* is important. One is in the statement of condition 4. The second is in the proof of that condition 2 implies condition 3. However, since condition 2 does not depend on the order of the vertices, but on the graph structure, we may characterize threshold digraphs in the absence of the condition that *α* is in positive lexicographic order. In particular, condition 3 gives that the digraph is threshold even when the degree sequence is unordered.

**Corollary 2.** *Let G be a digraph and A* = [*a_ij_* ] *an adjacency matrix of G. Define* $\alpha_{i}^{+}=\sum_{j=1}^{n}a_{ij}$ *and* $\alpha_{j}^{{}^{-}}=\sum_{i=1}^{n}a_{ij}$*. If for every triple of distinct indices i, j and k with i* < *j and a_jk_* = 1,* it also holds that a_ik_* = 1,* then G is a threshold digraph.*

*Proof.* We show that such a graph cannot have 2-switches or induced directed 3-cycles. A 2-switch is formed with four distinct indices, *i*, *j, k* and *l* so that *a_ij_* = *a_kl_* = 1 and *a_il_* = *a_kj_* = 0. Without the loss of generality, suppose that *i* < *k.* If condition 3 holds, then *a_kl_* = 1 gives *a_il_* = 1, so there are no 2-switches. Similarly, an induced directed 3-cycle is formed with three distinct indices, *i, j* and *k* so that *a_ij_* = *a_jk_* = *aki* = 1 and *a_ik_* = *a_kj_* = *a_ji_* = 0. Suppose that *i* is the smallest of the three indices. If condition 3 holds and *a_ik_* = 1, then *a_ik_* = 1 so we cannot have an induced directed 3-cycle, either.

Corollary 2 gives us a constructive method for creating threshold digraphs.

**Corollary 3.** *Given a sequence β* = (*β*_1_,…,*β_n_*), * with* 0 ≤ *β_j_* < *n for all j, if we define an n*× *n matrix A* = [*a_ij_* ] *by*

(3) $$a_{ij}=\{\begin{array}{ll} 1 & i<j\;\text{and}\;i\leq \beta_{j}\\ 1 & i>j\;\text{and}\;i\leq \beta_{j}+1\\ 0 & \text{otherwise}, \end{array}$$

then the matrix *A* is the adjacency matrix of a threshold digraph. Furthermore, if *G* is a threshold digraph and $\alpha =((\alpha_{1}^{+},\alpha_{1}^{{}^{-}}),\ldots ,(\alpha_{n}^{+},\alpha_{n}^{{}^{-}}))$, then the sequence $\beta =(\alpha_{1}^{{}^{-}},\ldots ,\alpha_{n}^{{}^{-}})$ generates an adjacency matrix of *G.*

*Proof.* Since *A* satisfies condition 3, Corollary 2 gives that it is threshold. For a threshold digraph *G*, the only matrix which satisfies both condition 3 and the condition $\sum_{i=1}^{n}a_{ij}=\alpha_{j}^{{}^{-}}$ is the matrix formed as above. Thus, *A* must be the adjacency matrix of *G*.

Since Corollary 3 ties together sequences and threshold digraphs, one application of it is to provide upper and lower bounds on the number of threshold digraphs for a given *n*. However, if we permute a sequence, then the resulting threshold digraph may or may not be isomorphic. For example, on three vertices the six orders of the sequence (2,1,0) produce two non-isomorphic threshold digraphs. The sequences (2,1,0), (1,2,0), and (2,0,1) all produce the same digraph with degree sequence ((1,2),(1,1),(1,0)) in positive lexicographic order, while the remaining three sequences produce the threshold digraph with degree sequence ((2,0),(1,1),(0,2)) in positive lexicographic order.

**Corollary 4.** *Define TD*(*n*) *as the number of threshold digraphs on n vertices. Then* $\frac{n^{n}}{n!}\leq TD(n)\leq n^{n}$.

## 3. Digraph Realizability

The idea of condition 4 comes from what are known as the Fulkerson-Chen inequalities for digraph realizability. Fulkerson studied digraph realizability in the context of zero-one matrices with zero trace [7]. For a given degree sequence, Fulkerson gave a system of 2*^n^* −1 inequalities that are satisfied if and only if the degree sequence is digraphical. The formulation that we typically use is due to Chen [8], which reduces the number of inequalities from 2*^n^* −1 to *n* when the degree sequence is in negative lexicographic order. Our consideration of threshold digraphs gives a new proof of this result.

This proof uses the partial order ≼, commonly called **majorization** [9], on integer sequences. In particular, for sequences *α* = (*α*_1_,…,*α_n_*) and *β* = (*β*_1_,…,*β*_n_) we say *α* ≼ *β* if $\sum_{i=1}^{k}\alpha_{i}\leq \sum_{i=1}^{k}\beta_{i}$ for *k* = 1,…,*n* −1 and $\sum_{i=1}^{n}\alpha_{i}=\sum_{i=1}^{n}\beta_{i}$. One important property of this partial order is that if *α* ≠ *β* and *α* ≼ *β*, then there is an index *i* such that *α_i_* < *β_i_* and a first index *j* > *i* with $\sum_{k=1}^{j}\alpha_{k}=\sum_{k=1}^{j}\beta_{k}$.

**Theorem 5.** *Let* $\alpha =((\alpha_{1}^{+},\alpha_{1}^{{}^{-}}),\ldots ,(\alpha_{n}^{+},\alpha_{n}^{{}^{-}}))$ *be a degree sequence in positive lexicographic order. There is a digraph G which realizes α if and only if* $\sum \alpha_{i}^{+}=\sum \alpha_{i}^{{}^{-}}$ *and for every k with* 1 ≤ *k* < *n*

(4) $$\sum_{i=1}^{k}\min(\alpha_{i}^{{}^{-}},k-1)+\sum_{i=k+1}^{n}\min(\alpha_{i}^{{}^{-}},k)\geq \sum_{i=1}^{k}\alpha_{i}^{+}.$$

*Proof.* Suppose that *G* realizes *α* with adjacency matrix *A*. Define

(5) $$c(i,k)=|\{j\leq k|a_{ji}=1\}|$$

as in the proof of Theorem 1, we see that

(6) $$\sum_{i=1}^{k}\alpha_{i}^{+}=\sum_{i=1}^{n}c(i,k)\leq \sum_{i=1}^{k}\min(\alpha_{i}^{{}^{-}},k-1)+\sum_{i=k+1}^{n}\min(\alpha_{i}^{{}^{-}},k),$$

as desired.

Suppose that *α* is a sequence which satisfies the above inequalities. Construct an adjacency matrix *T* as in Corollary 3 from the sequence *α^−^*. We will iteratively form a sequence of digraphs $T=B^{\left(0\right)},B^{\left(1\right)},\ldots ,B^{\left(t_{\max}\right)}$ with $B^{\left(t_{\max}\right)}$an adjacency matrix realizing *α*, with *β*^(^*^t^*^)^ the sequence of row sums in the matrix *B*^(^*^t^*^)^. By hypothesis, *α*^+^ ≼β ^(0)^. If *α* ^+^ = *β*^(0)^, then *t*_max_ = 0 and *T* = *B*^(0)^ is the adjacency matrix of the desired graph. Otherwise, define $t_{\max}=\frac{1}{2}\sum_{i=1}^{n}|\alpha_{i}^{+}-\beta_{i}^{\left(0\right)}|$, and let *r*(1,*t*) and *r*(2,*t*) be indices such that *r*(1,*t*) is the smallest index where $\alpha_{r(1,t)}^{+}<\beta_{r(1,t)}^{\left(t\right)}$ and *r*(2,*t*) the first index after *r*(1,*t*) such that $\sum_{i=1}^{r(2,t)}\alpha_{i}^{+}=\sum_{i=1}^{r(2,t)}\beta_{i}^{\left(t\right)}$. For *t*< *t*_max_, define $\beta^{\left(t+1\right)}=(\beta_{1}^{\left(t+1\right)},\ldots ,\beta_{n}^{\left(t+1\right)})$ as the sequence with

(7) $$\beta_{i}^{\left(t+1\right)}=\{\begin{array}{ll} \beta_{i}^{\left(t\right)}-1 & i=r(1,t)\\ \beta_{i}^{\left(t\right)}+1 & i=r(2,t)\\ \beta_{i}^{\left(t\right)} & \text{otherwise}. \end{array}$$

Clearly *β*^(^*^t^*^)^ ≻ *β*^(^*^t^*^+1)^ ≽ *α*^+^. Since $\alpha_{r(2,t)}^{+}-1\geq \beta_{r(2,t)}^{\left(t\right)}$ and $\alpha_{r(1,t)}^{+}+1\leq \beta_{r(1,t)}^{\left(t\right)}$, we have

(8) $$\beta_{r(1,t)}^{\left(t\right)}-\beta_{r(2,t)}^{\left(t\right)}\geq (\alpha_{r(1,t)}^{+}+1)-(\alpha_{r(2,t)}^{+}-1)\geq 2.$$

Thus, there are columns *c*(1,*t*) and *c*(2,*t*) of *B*^(^*^t^*^)^ that have ones in row *r*(1,*t*) and zeros in row *r*(2,*t*). Either *c*(1*t*) ≠ *r*(2,*t*) or *c*(2,*t*) ≠ *r*(2,*t*); therefore, without the loss of generality, we may suppose that *c*(1, *t*) ≠ *r*(2, *t*). Let *B*^(^*^t^*^+1)^ be the matrix with

(9) $$b_{ij}^{\left(t+1\right)}=\{\begin{array}{ll} 0 & i=r(1,t),j=c(1,t)\\ 1 & i=r(2,t),j=c(1,t)\\ b_{ij}^{\left(t\right)} & \text{otherwise}. \end{array}$$

Since $\sum_{i=1}^{n}\left|\alpha_{i}^{+}-\beta_{i}^{\left(t+1\right)}\right|=\sum_{i=1}^{n}|\alpha_{i}^{+}-\beta_{i}^{\left(t\right)}|-2$, we have that

(10) $$\sum_{i=1}^{n}\left|\alpha_{i}^{+}-\beta_{i}^{\left(t_{\max}\right)}\right|=\sum_{i=1}^{n}\left|\alpha_{i}^{+}-\beta_{i}^{\left(0\right)}\right|-2t_{\max}=0.$$

Therefore, $\beta^{\left(t_{\max}\right)}=\alpha^{+}$ and $B^{\left(t_{\max}\right)}$ is a realization of *α*, as desired.

This proof is constructive; given a digraphical degree sequence *α*, we can construct a realization of *α* by repeatedly moving the ones down in the columns as in the proof of Theorem 5. There are other construction algorithms for digraphs, most notably that of Kleitman and Wang [10].

## 4. Applications

What follows is a quick survey of some consequences of Theorem 1. Some details are omitted since the first two results are immediate.

Threshold graphs, in the undirected sense, are closely tied to the theory of split graphs. An analogous study of **split digraphs** is given by LaMar [11]. Using the fourth characterization of threshold digraphs and a result by LaMar, the immediate implication is Corollary 6.

**Corollary 6.** *Every threshold digraph is a split digraph.*

Merris and Roby [12] studied the relationship between different threshold graphs as subgraphs of one another. As a simple consequence of the third characterization of threshold digraphs, there is a similar relationship between threshold digraphs which we state as Corollary 7.

**Corollary 7.** *Given a threshold digraph G, if G is nonempty, then there is an arc e in G such that G* − *e is a threshold digraph. If G is not complete, then there is an arc e not in G such that G* + *e is a threshold digraph.*

It has been observed that the ordering required by Theorem 5 can be relaxed and still only require the *n* inequalities stated. Berger [4] observed that we need only require nonincreasing order in the first component. Our theorem suggests that this can be relaxed even more, but it is not readily apparent which orders should be considered for graphicality. However, we can show a simple proof that nonincreasing order in the first component is sufficient.

**Theorem 8.** *Let α be an integer pair sequence satisfying* $\alpha_{i}^{+}\geq \alpha_{i+1}^{+}$ *for every* 1 ≤ *i* < *n*. *If* $\sum \alpha_{i}^{+}=\sum \alpha_{i}^{{}^{-}}$ *and*

(11) $$\sum_{i=1}^{k}\min(\alpha_{i}^{{}^{-}},k-1)+\sum_{i=k+1}^{n}\min(\alpha_{i}^{{}^{-}},k)\geq \sum_{i=1}^{k}\alpha_{i}^{+}$$

for 1 < *k* < *n*, then *α* is digraphical.

*Proof.* If *α* is in positive lexicographic order, then this is true by Theorem 5. Otherwise, let *l* be an index so that $\alpha_{l}^{+}=\alpha_{l+1}^{+}$ and $\alpha_{l}^{{}^{-}}<\alpha_{l+1}^{{}^{-}}$. Form the integer pair sequence *β* from *α* by exchanging $\alpha_{l}^{{}^{-}}$ and $\alpha_{l+1}^{{}^{-}}$. We show that *α* satisfies all the inequalities if and only if *β* satisfies all the inequalities.

From *α*^−^, form the matrix *A* as in Corollary 3 and let *s_i_* be the row sums in *A*. From *β*^−^, form the matrix *B* and let *s_i_* be the row sums in *B*. Notice

(12) $$\sum_{i=1}^{k}\min(\alpha_{i}^{{}^{-}},k-1)+\sum_{i=k+1}^{n}\min(\alpha_{i}^{{}^{-}},k)=\sum_{i=1}^{k}s_{i}$$

and a similar equality holds for the sums $\sum_{i=1}^{k}s_{i^{\prime }}$.

Notice that *A* and *B* differ only in the columns *l* and *l* +1. Consider the entries in columns *l* and *l* +1. We have *a_i,l_* = *b_i,l_*_+1_ and *a_i,l_*_+1_ = *b_i,l_* for every *i* ∉{*l*,*l* +1}; therefore, the row sums are equal except at these two indices. If *a_l,l_*_+1_ = *a_l_*_+1,_*_l_*, then $s_{l}=s_{l}^{\prime }$ and $s_{l+1}=s_{l+1}^{\prime }$ ; therefore, since *s* and *s′* are the same sequence, we have that $\sum_{i=1}^{k}s_{i}\geq \sum_{i=1}^{k}\alpha_{i}^{+}$ if and only if $\sum_{i=1}^{k}s_{i^{\prime }}\geq \sum_{i=1}^{k}\alpha_{i}^{+}$. In general, we wish to show that $\sum_{i=1}^{k}s_{i}\geq \sum_{i=1}^{k}\alpha_{i}^{+}$ for all *k* if and only if $\sum_{i=1}^{k}s_{i^{\prime }}\geq \sum_{i=1}^{k}\alpha_{i}^{+}$ for all *k*.

Since $\alpha_{l}^{{}^{-}}<\alpha_{l+1}^{{}^{-}}$ it remains only to consider the case where *a_l,l_*_+1_ = 1 and *a_l_*_+1,_*_l_* = 0. In this case, the construction of *A* gives that *s_l_* > *s_l_*_+1_. We also have that *s_l_*_′_ = *s_l_* −1 and *s_l_*_+1_' = *s_l_*_+1_ +1, thus $\sum_{i=1}^{k}s_{i}=\sum_{i=1}^{k}s_{i^{\prime }}$ for every *k* ≠ *l* and $\sum_{i=1}^{l}s_{i}=\sum_{i=1}^{l}s_{i^{\prime }}+1$. Therefore, for *k* < *l* or *k* > *l* +1, we have that $\sum_{i=1}^{k}s_{i}\geq \sum_{i=1}^{k}\alpha_{i}^{+}$ if and only if $\sum_{i=1}^{k}s_{i^{\prime }}\geq \sum_{i=1}^{k}\alpha_{i}^{+}$.

Since the sequences fail the inequalities 11 with *k* < *l* at the same time, and one failed condition is enough to not pass this graphicality test, we assume that $\sum_{i=1}^{k}s_{i}=\sum_{i=1}^{k}s_{i^{\prime }}\geq \sum_{i=1}^{k}\alpha_{i}^{+}$ for *k* < *l*. The only way to have exactly one of the inequalities 11 fail at *k* = *l* is if $\sum_{i=1}^{l}s_{i^{\prime }}<\sum_{i=1}^{l}\alpha_{i}^{+}$ and $\sum_{i=1}^{l}s_{i}\geq \sum_{i=1}^{l}\alpha_{i}^{+}$. Thus, $\sum_{i=1}^{l}s_{i}=\sum_{i=1}^{l}\alpha_{i}^{+}$ and $s_{l}\leq \alpha_{l}^{+}$. Both *α* and *β* fail at least one condition since

(13) $$\alpha_{l+1}^{+}=\alpha_{l}^{+}\geq s_{l}>s_{l+1}$$

implies that $\sum_{i=1}^{l+1}\alpha_{i}^{+}>\sum_{i=1}^{l+1}s_{i}$.

This section gives a brief overview of some of the applications of threshold digraphs. The uses of threshold graphs in various disciplines has been studied extensively, as shown in Mahadev and Peled’s text [3]. These results are a starting point for an analogous study of threshold digraphs.
